# Mechanism of Growth Phase-Dependent Nanoplastic Bioaccumulation in *Tetrahymena thermophila*

**DOI:** 10.3390/antiox14121456

**Published:** 2025-12-04

**Authors:** Zhongquan Jiang, Tianyi Wei, Haipeng Tong, Ruikai Xing, Di Peng, Tao Yuan, Ling Zhao, Minghua Min, Wenbo Guo

**Affiliations:** 1 East China Sea Fisheries Research Institute, Chinese Academy of Fishery Sciences, Shanghai 200090, China; zhongquanj@sjtu.edu.cn (Z.J.); pengdi@ecsf.ac.cn (D.P.); 2 Key Laboratory of Environmental Health Impact Assessment of Emerging Contaminants, Ministry of Ecology and Environment, School of Environmental Science and Engineering, Shanghai Jiao Tong University, Shanghai 200240, China; sardine0722@sjtu.edu.cn (T.W.); tong-arabidopsis@sjtu.edu.cn (H.T.); xrk382937427@sjtu.edu.cn (R.X.); taoyuan@sjtu.edu.cn (T.Y.); wszhaoling@sjtu.edu.cn (L.Z.); 3 Zhoushan Putuo Deep Blue Fishery Technology Research and Development Center, Zhoushan 316100, China; 4 International Research Center for Marine Biosciences, Ministry of Science and Technology, College of Fisheries and Life Science, Shanghai Ocean University, Shanghai 201306, China

**Keywords:** nanoplastics, protozoan, bioaccumulation, oxidative stress, proteomics

## Abstract

Nanoplastics are ubiquitous in aquatic environments, and elucidating their bioaccumulation behavior is essential for assessing toxicity and trophic transfer risks. While most studies focus on nanoplastics properties (e.g., type, size, surface charge), the influence of organismal growth stage remains unclear. Through bioaccumulation kinetic experiments with 10 mg/L polystyrene nanoplastics (PSNPs), this study found that *Tetrahymena thermophila* (*T. thermophila*) in the lag phase (cell density 4 × 10^4^ cells/mL) exhibited the highest uptake rate of nanoplastics, 1.2–5.8 times that of the exponential phase (1–5 × 10^5^ cells/mL) and 7.7 times that of the stationary phase (>5 × 10^5^ cells/mL). Lag phase cells also had a larger specific surface area (0.319 vs. 0.271/0.269 μm^−1^), supporting their heightened uptake capacity. Under PSNP exposure, exponential and stationary phase cells showed significantly elevated reactive oxygen species (ROS) levels, accompanied by downregulated superoxide dismutase (SOD) and stable catalase (CAT) activity, indicating impaired antioxidant defense and potential redirection of energy toward stress mitigation. Consistent with efficient internalization, confocal imaging revealed clear PSNP colocalization within food vacuoles of lag period cells. Proteomic and transcriptomic analysis further confirmed the upregulation of carrier proteins, FAD/FMN oxidoreductases, and pathways associated with cellular components (membrane and organelle membrane) and molecular functions (transporter activity and transmembrane transporter activity) in lag-phase *T. thermophila*. Collectively, these findings provide a molecular-level understanding of the multi-phase-dependent bioaccumulation of PSNPs, offering critical insights for assessing the environmental risks of polystyrene nanoplastics in dynamic aquatic ecosystems.

## 1. Introduction

Nanoplastics (NPs), typically defined as minute particles with a diameter smaller than 1 µm, are widely recognized as the most challenging component of global plastic pollution [1]. NPs are ubiquitous in aquatic ecosystems, with their sources broadly categorized into two types: first, the degradation of larger microplastics through environmental weathering, industrial discharges, and urban activities; and second, direct contributions from fishery operations, such as discarded nets and gear or the degradation of aging fishing buoys. These latter sources significantly contribute to the enrichment of NPs in water bodies [2,3]. The size effect of nanoparticles confers unique environmental risks. On one hand, NPs possess a high specific surface area and reactivity [4], enabling them to act as effective vectors for other pollutants, thereby influencing the fate and toxicity of these contaminants [5]. On the other hand, NPs can readily penetrate biological barriers, including cell membranes and the blood–brain barrier [6,7]. This leads to their accumulation within organisms and distribution to other tissues and organs, causing damage to biological tissues and organs or even individual mortality [8]. Bioaccumulation serves as the critical link between the environmental exposure levels of NPs and their subsequent ecotoxicological effects [9]. It also provides the foundation for the trophic transfer of NPs up the food web [10]. However, current research on NP bioaccumulation has predominantly focused on NP physicochemical properties (e.g., size, shape, surface charge) [11,12]. The physiological dynamics and growth cycles of exposed organisms are largely overlooked, which is a critical gap as microbial growth phases modulate cellular uptake and metabolic responses.

In eukaryotic cells, NP uptake is a highly regulated, energy-dependent process (endocytosis for 1–100 nm particles; phagocytosis for aggregated NPs > 500 nm) [13,14], with efficiency closely linked to cellular physiological state. Critically, NP size and biological barrier penetration—key factors in bioaccumulation—are modulated by growth phase-dependent changes in cell membrane permeability and endocytic activity. For example, cellular uptake capacity varies with the cell cycle (G2/M > S > G0/G1) due to differences in cell division rates and intracellular transport [15,16], highlighting the potential for growth phase-dependent bioaccumulation dynamics. The cell’s metabolic rate and cell cycle stage—a series of events within individual cells leading to division (G1, S, G2/M phases)—directly govern the energy supply required for these membrane dynamics and cytoskeletal activities [15]. In contrast, the microbial growth cycle (lag, exponential, stationary phases) describes population-level growth dynamics, reflecting distinct collective physiological and metabolic states of the cell community. For heterotrophic protozoa like *Tetrahymena thermophila* (*T. thermophila*), which rely primarily on phagocytosis to acquire particulate nutrients [17], abundant environmental nutrients are known to induce changes in feeding and clearance behaviors [18]. If NPs are non-selectively incorporated into this phagocytic pathway, this enhanced particle uptake could lead to a maximum accumulation rate for non-nutritive NPs. Therefore, variations in metabolic priorities and energy allocation are key biodynamic factors regulating NP accumulation.

*T. thermophila* is an ideal model for investigating growth phase-dependent NP bioaccumulation. As a standardized model protozoan by ISO 2022 (https://www.iso.org/standard/80595.html) [19] with conserved eukaryotic cellular processes, findings can be extrapolated to other microbial eukaryotes in aquatic ecosystems [20,21]. Ecologically, *T. thermophila*’s role as a primary consumer in microbial food webs ensures ecological relevance to NP trophic transfer risks [22]. Of particular importance, *T. thermophila*’s primary uptake route for particulate matter is endocytosis via a specialized oral apparatus, a process known to be modulated by growth phase-specific metabolic activity, making it suitable for studying phase-dependent NP internalization [23]. Furthermore, *T. thermophila* exhibits distinct, well-characterized lag, exponential, and stationary phases in batch culture, enabling precise separation and analysis of growth-dependent bioaccumulation mechanisms [24]. These features allow direct investigation of how cellular physiological states (linked to growth phases) regulate NP endocytosis dynamics, particularly the hypothesis that lag phase cells may exhibit unique bioaccumulation mechanisms due to metabolic priming.

This study aims to determine whether significant differences exist in the bioaccumulation rates of polystyrene nanoplastics (PSNPs) in *T. thermophila* across its different growth cycles, and to elucidate the key physiological regulatory mechanisms driving these potential differences. We established standardized cell models for the lag, exponential, and stationary phases by controlling the *T. thermophila* culture conditions. By using green fluorescently labeled PSNPs, we systematically quantified and compared the short-term accumulation kinetics in *T. thermophila* at these different growth stages. We further integrated oxidative stress, antioxidant-related parameters and proteomic analyses to investigate the physiological mechanisms governing the differential accumulation rates. Previous studies have primarily been conducted under conditions of optimized nutrition (simulating the exponential growth phase), which may systematically underestimate the true accumulation risks of PSNPs in organisms within more complex natural environments. Therefore, the core significance of this study lies in its innovative approach of incorporating the dynamic microbial growth cycle as a key variable in the risk assessment framework for PSNP bioaccumulation. This work provides an ecologically relevant theoretical foundation for a more accurate assessment of NP ecological risks in natural scenarios.

## 2. Materials and Methods

### 2.1. T. thermophila Culture and Chemicals

The protozoan *T. thermophila* SB210 (50 × 20 µm) was purchased from the Institute of Hydrobiology, Chinese Academy of Sciences. *T. thermophila* was routinely maintained in sterile SPP (Super Proteose Peptone) medium at 25 °C. SPP is a nutrient-rich medium composed of 2% (*w*/*w*) proteose peptone, 0.1% (*w*/*w*) yeast extract, 0.2% (*w*/*w*) glucose, and 0.003% (*w*/*w*) ferric citrate (Table S1). To ensure sterility, the medium was supplemented with 100 units/mL penicillin G, 100 μg/mL streptomycin sulfate, and 0.025 μg/mL amphotericin B. All nanoparticle exposure experiments were conducted in Dryl’s medium (pH 6.8–7.0). This minimal salt solution consists of 2 mM NaH_2_PO_4_·H_2_O, 1 mM Na_2_HPO_4_, and 1.5 mM CaCl_2_ (Table S2). Dryl’s medium was used to prevent organic matter from the SPP medium from binding with the NPs, which could interfere with NP aggregation status and the cellular endocytosis process.

**Figure 1:**
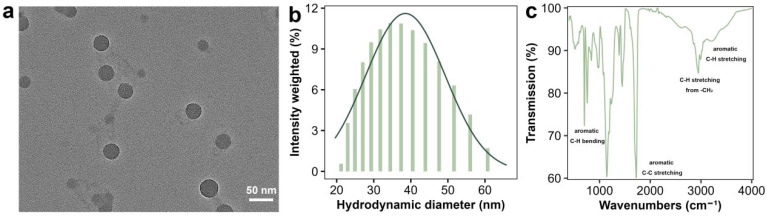
Characterization of PSNPs. Transmission electron microscopy image (**a**) and size distribution of the PSNP particles (**b**). Fourier transform infrared spectroscopy of PSNPs (**c**). The data in (**b**) represents the average of five measurements.

Green fluorescent-labeled PSNPs (2.5% *w*/*v* aqueous suspension) were purchased from YUAN BIOTECH (Shanghai, China). The morphology of PSNPs was examined using transmission electron microscopy (TEM, Tecnai G2 Spirit TWIN, Aarhus, Denmark, Thermo Fisher Scientific, Waltham, MA, USA) (Figure 1a). The hydrodynamic diameter and zeta potential of PSNPs were measured via dynamic light scattering (DLS, ZetaPALS, Brookhaven Instruments, Holtsville, NY, USA) while resuspended in the experimental medium (Figure 1b). The high colloidal stability (zeta potential < −25 mV, PDI < 0.2) indicates strong electrostatic repulsion, which prevents aggregation or sedimentation over the short exposure duration (1.5 h). Cultures were gently agitated every 15 min to further minimize potential settling. The chemical structure of PSNPs was determined using Fourier transform infrared spectroscopy (FTIR) (Thermo Fisher Scientific, Nicolet 6700) (Figure 1c).

### 2.2. Acute Toxicity and Oxidative Stress Assays

Before conducting the bioaccumulation kinetics study, the acute toxicity of PSNPs to *T. thermophila* was determined. *T. thermophila* in the lag, exponential, and stationary growth phases were washed twice with Dryl’s and resuspended in Dryl’s medium containing 0, 0.1, 1, 10, and 100 mg/L of PSNPs, with a cell concentration of 1 × 10^5^ cells/mL (for cumulative kinetics experiments). After 24 h of exposure, cell density in each treatment group was monitored using the Countess 3 Automated Cell Counter (Thermo Fisher Scientific, USA).

The toxicity of PSNPs to *T. thermophila* at different growth stages was assessed by measuring the production of reactive oxygen species (ROS), superoxide dismutase (SOD), and catalase (CAT). Negative controls were cells without PSNP exposure, positive control were cells treated with 100 μM H_2_O_2_. Intracellular ROS levels were measured using the 2′,7′-dichlorodihydrofluorescein diacetate (DCFH-DA) assay kit (Beyotime Biotechnology Inc., Shanghai, China). SOD activity in *T. thermophila* cells was determined using the nitroblue tetrazolium (NBT) colorimetric method with the total SOD activity assay kit. CAT levels in *T. thermophila* cells were quantified using the hydrogen peroxide assay kit based on the N-(4-antipyryl)-3-chloro-5-sulfonate-p-benzoquinonemonoimine colorimetric reaction. While some studies suggest that markers such as GSH or MDA could provide additional insights, SOD and ROS are considered appropriate and complementary approaches for monitoring superoxide-related stress during short-term exposure. Detailed experimental procedures can be found in the Supplementary Information (SI, Text S1). All treatment groups included three biological replicates, and statistical analysis was performed following the methodology outlined in Section 2.7.

### 2.3. PSNPs Uptake by Different Growth Phases of T. thermophila

To minimize experimental variation and ensure concurrent measurement, *T. thermophila* cultures were inoculated at staggered time points based on a pre-established standard growth curve. This design allowed for the simultaneous assessment of PSNP bioaccumulation in cells harvested from different growth phases, with explicit time windows to avoid overlap. The following key physiological stages were defined for sampling and exposure: Lag phase: cell density approximately 1 × 10^4^–1 × 10^5^ cells/mL; exponential phase: cell density approximately 1 × 10^5^–5 × 10^5^ cells/mL; stationary phase: cell density approximately ≥ 5 × 10^5^ cells/mL. *T. thermophila* cells from each defined phase were harvested, washed three times with Dryl’s medium, and subsequently resuspended in 15 mL of pre-equilibrated Dryl’s medium containing 10 mg/L PSNPs. Considering that the concentration range of PSNPs selected in relevant studies typically spans from 1 to 300 mg/L, the concentration of PSNPs was set at 10 mg/L in the present study to meet the requirements for investigating accumulation kinetics [25]. After resuspension, the cell concentration at each stage was maintained at 1–1.2 × 10^5^ cells/mL. To ensure a constant temperature during sampling, all treatment groups were placed in a water bath at 25 °C. At time points 0.25, 0.5, 1, and 1.5 h, 1 mL aliquots were collected, and the single-cell fluorescence intensity was measured using flow cytometry (BD LSRFortessa, San Jose, CA, USA).

The accumulation data were fitted using a pseudo-first-order kinetic model (Formulas (1)–(3)) to determine the PSNPs uptake rate constant $k_{u}$ and the cell surface adsorption (represented by the y-intercept). In the general Formula (1), $k_{u}$ represents the uptake rate constant (L/cell/h); ${\left[\mathrm{NPs}\right]}_{\mathrm{med}}$ is the concentration of PSNPs in the medium (mg/L); $k_{e}$ is the rate constant for the elimination of intracellular PSNPs (/h); and µ is the specific growth rate of the cells (/h).

(1) $$\frac{{d\left[\mathrm{NPs}\right]}_{\mathrm{cell}}}{dt}={\;k}_{u}\;\times \;{\left[\mathrm{NPs}\right]}_{\mathrm{med}}\;-\;\left(k_{e}+\mu \right)\;\times \;{\left[\mathrm{NPs}\right]}_{\mathrm{cell}}$$

For the short-term uptake experiments, $k_{e}$ and µ were assumed to be 0 (negligible). This simplification is widely accepted in *T. thermophila* uptake studies, as excretion is minimal over short durations and cell growth during exposure is <5% (confirmed by cell count). At this point, the simplified formula (Formula (2)) was used for fitting:

(2) $$\frac{{d\left[\mathrm{NPs}\right]}_{\mathrm{cell}}}{dt}\;={\;k}_{u}\;\times \;{\left[\mathrm{NPs}\right]}_{\mathrm{med}}$$

Integrating Formula (2) yields Formula (3).

(3) $${\left[\mathrm{NPs}\right]}_{\mathrm{cell}}=k_{u}\;\times \;{\left[\mathrm{NPs}\right]}_{\mathrm{med}}\;\times \;t+C$$

In Formula (3), the constant C represents the initial adsorption of PSNPs onto the organism at t = 0. It should be noted that this model was used to compare the relative uptake rates between different stages, rather than to quantify accumulation kinetic parameters, which is consistent with its application in similar studies [25]. Furthermore, while a two-compartment model or single-cell sorting could explicitly address issues of bioaccumulation and efflux, such approaches were beyond the scope of this study. At the selected accumulation duration, nanoparticle efflux by the organisms is considered negligible. Since the primary focus of this work was to determine growth stage-dependent trends in nanoparticle accumulation rates, the application of a pseudo-first-order kinetic model was deemed appropriate. The uptake rate constant $k_{u}$ can be calculated from the intracellular PSNP accumulation at specific times and the exposure concentration. Linear regression was performed using the lm() function in R (version 4.5.1). All growth stage models demonstrated a strong fit, with r^2^ > 0.9.

### 2.4. High-Resolution Microscopy Imaging

To visually observe the bioaccumulation of PSNPs in *T. thermophila* at different growth stages, cells were collected after 1 h of PSNP exposure and washed once with Dryl’s solution. Negative controls are cells without PSNP exposure, to exclude autofluorescence, and positive control are cells treated with fluorescent latex beads of known uptake efficiency. The cell membranes were stained with Dil (excitation/emission wavelengths: 549/565 nm, BeyoTech Biotechnology Co., Shanghai, China) at 28 °C for 0.5 h. The cells were then centrifuged, washed, and resuspended in 100 μL of Dryl’s solution. A 5 μL sample from the suspension was placed on a glass slide and immediately imaged using a Leica high-resolution confocal laser scanning microscope (STELLARIS 5, Wetzlar, Germany). The excitation light was adjusted to optimize focus and a 488 nm (approximately 50 mW) laser source, and fluorescence was observed with a 100 × 1.45 NA oil immersion objective. Images were captured in a 512 × 512-pixel area on an EMCCD camera. Three-dimensional images were acquired with a z-step of 0.2 μm, resulting in a total thickness of 3.3 μm. The images were analyzed using Leica Application Suite X (Version 3.7.23463.4, Leica, Wetzlar, Germany) or Fiji software (Version 2.16.0, NIH, Bethesda, MD, USA).

### 2.5. Proteomic and Transcriptomic Analysis

Proteomic analysis was performed to elucidate the differences in endocytosis and protein functions at different growth stages of *T. thermophila*. Quality control (QC) samples are pooled protein extracts to ensure instrument stability. For protein extraction, cells were lysed on ice in a lysis buffer containing a protease inhibitor (PMSF) and a detergent (SDS). The total protein concentration was accurately determined using the BCA (Bicinchoninic Acid) assay. Subsequently, proteins were reduced (with DTT) and alkylated (with IAM) to cleave and block disulfide bonds, followed by digestion into peptides using trypsin. The peptides were then desalted using chromatography on an SDB column. Quantitative proteomics was performed using either a Label-Free Quantification (LFQ) or a TMT (Tandem Mass Tags) labeling technique. The TMT technique enables relative protein quantification between samples based on the abundance of its reporter ions. To increase detection depth, the peptide mixture was fractionated by HPLC (High-Performance Liquid Chromatography), typically into 10–30 fractions. The proteomic sample preparation and analytical workflow consisted of four main steps: protein extraction/quality control, enzymatic digestion, peptide processing, and LC MS/MS detection followed by data analysis. These steps are briefly outlined as follows: (1) Protein Extraction and QC: Frozen samples were lysed, homogenized, and sonicated in ice-cold buffer containing protease inhibitors. After centrifugation, the supernatant was collected for protein quantification using the BCA assay. The precipitate was redissolved, sonicated, and centrifuged again, with the protein content measured via ProteoAnalyzer; (2) Enzymatic Digestion: 100 μg of protein was reduced with TCEP and alkylated with IAM in TEAB buffer. After centrifugation, the pellet was resuspended and digested with trypsin (1:50) at 37 °C overnight. The resulting peptides were dried, reconstituted in 0.1% TFA, desalted using HLB, and quantified by NanoDrop; (3) Peptide Handling and MS Detection: LC-MS/MS analysis was performed using a Vanquish Neo UHPLC system coupled to an Orbitrap Astral or timsTOF Ultra2 (Bruker, Germany) mass spectrometer. Separation was achieved with an 8 min gradient in DIA mode, and mass detection covered a scan range of 100–1700 *m*/*z*. (4) Data Analysis: Protein identification and quantification (MaxLFQ) were performed in Spectronaut. Statistical analysis (*t*-test for DEPs), GO annotation, and protein–protein interaction (PPI) analysis using STRING were conducted on the Majorbio Cloud Platform. More detailed procedures are provided in Text S2 of the SI.

Transcriptomic analysis was performed to identify gene expression variations across different growth stages of *T. thermophila*. Total RNA was isolated from cells at densities of 4 × 10^4^, 4 × 10^5^, and 1 × 10^6^ cells/mL (approximately 10^7^ cells per sample), with three biological replicates for each condition. Total RNA was extracted with TRIzol^®^ and quality-checked (OD260/280 = 1.8–2.2, RIN ≥ 6.5). Sequencing libraries were constructed from high-quality RNA (>1 μg) using the Illumina^®^ Stranded mRNA Prep kit (San Diego, CA, USA), followed by sequencing on NovaSeq X Plus or DNBSEQ-T7 platforms (PE150). After quality control with fastp, clean reads were aligned to the reference genome using HISAT2 and assembled with StringTie. Differential expression analysis (|log2FC| ≥ 1, FDR < 0.05) was performed with DESeq2/DEGseq, followed by functional enrichment (GO, KEGG) using Goatools and Python (Version 3.12) scipy [26,27]. Alternative splicing events were identified with rMATS. Detailed procedures are provided in Text S3 of the SI.

### 2.6. Molecular Docking

This section provides prediction and validation of the NP interaction mechanisms at the molecular structure level. Molecular docking and molecular dynamics (MD) simulations were used to study the interaction between PSNPs and their putative target protein, the TLR4/MD-2 complex (PDB ID: 2Z64). The target protein was identified based on the proteomic analysis, and its structure was retrieved from the PDB (Protein Data Bank). Molecular docking was performed using MOE (Version 2019.0102) (Molecular Operating Environment) software to predict the binding sites and affinity of the polystyrene (PS) ligand within the hydrophobic pocket of MD-2. Subsequently, the resulting complex was subjected to a 50 ns MD simulation using GROMACS (Version 2025.2) with the AMBER 03 force field. The simulation was conducted under physiological conditions (310 K) to evaluate how NPs binding induces the flipping of key residues and the bending of protein domains, thereby promoting receptor dimerization.

### 2.7. Statistical Analysis

All experimental data were analyzed using IBM SPSS Statistics 22.0 and GraphPad Prism 9.0. Quantitative data (ROS, SOD, CAT activity, intracellular PSNP concentration) were normalized to cell number to account for growth phase-dependent differences in cell size and metabolic state. Proteomic data were normalized to total peptide intensity. Normality was assessed via the Shapiro–Wilk test, and homogeneity of variance via Levene’s test. Student’s *t*-test was used for pairwise comparisons. One-way ANOVA followed by Tukey’s HSD post hoc test was used for multiple comparisons. Two-way ANOVA was employed to analyze the interactive effects of PSNP concentration and growth cycle stage. Statistical significance was set at *p* < 0.05. Error bars represent standard deviation (SD) of 3 biological replicates.

## 3. Results and Discussion

### 3.1. Physicochemical Properties of the PSNPs

The PSNPs used in this study were well-dispersed and a polydispersity index of 0.126, indicating good colloidal stability in Dryl’s. The hydrodynamic diameter of the PSNPs was 37.36 ± 2.1 nm (Figure 1b). DLS measurements were performed at 25 °C, detection angle = 90°, with 10 scans per sample (Brookhaven Instruments ZetaPALS). It is important to note that the DLS-measured hydrodynamic diameter includes the core nanoparticle, any surface coating, and the solvation layer, and is typically larger than the dry-state, core diameter measured by TEM (Figure 1a) [28]. Their morphology was examined by TEM, revealing uniformly spherical particles with average diameters of 34.33 ± 3.2 nm (Figure 1a). Furthermore, the negative zeta potentials (−28.53 mV) suggested strong electrostatic repulsion, contributing to their stable dispersion. This is consistent with the low aggregation state observed in TEM, suggesting that the particles may be stabilized against aggregation through surface ligands or charge repulsion. Furthermore, the Dryl’s medium, used for exposure, is a well-characterized minimal salt solution with consistent physicochemical properties (pH 6.8–7.0) that minimize NP aggregation. Given the short exposure duration and gentle agitation of cultures during the experiment, significant aggregation or sedimentation is unlikely—consistent with low PDI and negative zeta potential of PSNPs in Dryl’s medium. Although direct stability measurements during incubation would provide additional insights, they are not critical in this context, as the consistent linear uptake kinetics (r^2^ > 0.9) confirm that PSNP remained bioavailable to the cells throughout the experimental period. The FTIR results confirmed the chemical composition of the PSNPs (Figure 1c). The absorption peak at 3430 cm^−1^ corresponds to O–H stretching vibrations, likely originating from the labeled fluorescent dye. Based on our previous study, dialysis (MWCO = 10 kDa, 24 h) was employed to ensure no leaching of fluorophores from the PSNPs used in the experiments. The carbon content of the dialysate was measured to confirm that the fluorescence signals in the uptake experiments reflected genuine cellular accumulation of PSNPs rather than free dye contamination. Absorption peaks at 3082, 3060, and 3025 cm^−1^ are characteristic of aromatic ring C–H stretching vibrations, while the peak at 2920 cm^−1^ is attributed to the asymmetric stretching vibration of aliphatic methylene groups. The absorption peaks at 1490 and 1450 cm^−1^ correspond to aromatic ring C=C skeletal stretching vibrations, and the peaks at 756 and 698 cm^−1^ are associated with aromatic ring C–H out-of-plane bending vibrations [29,30].

### 3.2. PSNP Uptake by T. thermophila as Affected by Growth Stage

**Figure 2:**
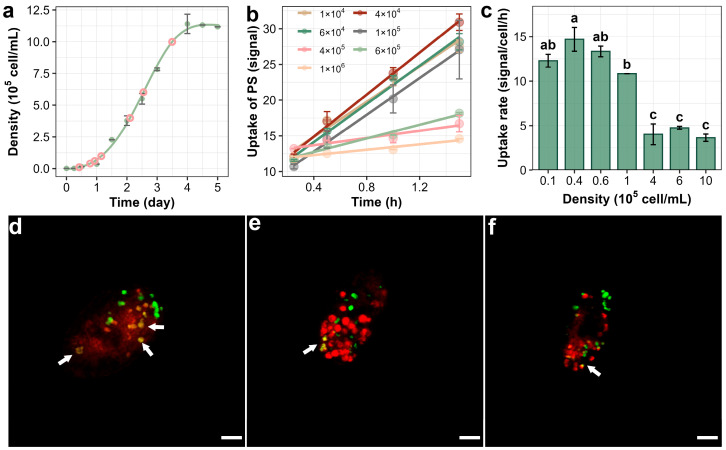
Bioaccumulationof PSNPs in *T. thermophila* during different growth phases. The growth curve of *T. thermophila* cells in nutrient-enriched medium (SPP) (**a**). The time-dependent increase in PSNPs in different growth phases of *T. thermophila* cells after exposure to 10 mg/L of PSNPs during a 1.5 h uptake period (**b**). Uptake rates of PSNPs in *T. thermophila* at different growth phases calculated from the bioaccumulation kinetics curves, different lowercase letters (a, b, c) and combined letters (ab) above the error bars indicate significant differences among groups (*p* < 0.05) (**c**). Data are the mean ± standard deviation (*n* = 3). Confocal microscopy images showing the accumulation of PSNPs in *T. thermophila* cells during the lag (**d**), exponential (**e**), and stationary (**f**) phases. In figure (**a**), the pink circles indicate the sampling time points for the kinetic experiment, and the green curve represents the cell density measured at the corresponding time points. In figure (**d**–**f**), the red signal indicates the food vacuole membrane, and the green signal denotes the PSNPs. Co-localization of PSNPs within the food vacuoles is highlighted with arrows. Scale bar = 10 μm.

The S-shaped curve describes how the population size gradually increases and stabilizes over time in a resource-limited environment [31]. According to Figure 2a, the growth dynamics of *T. thermophila* follow a classic S-shaped curve, where the cell population experiences a lag phase of approximately one day before reaching a stationary phase around day 4, with an exponential growth phase occurring between days 1 and 4. Using this growth curve, *T. thermophila* cells were inoculated at different time points to ensure that cells in various growth phases were obtained simultaneously.

Prior to conducting the bioaccumulation assay, the cytotoxic effects of PSNPs were determined to prevent potential concentration artifacts resulting from cell mortality. As shown in Figure S1, exposure to PSNPs at concentrations as high as 100 mg/L for 24 h did not induce significant changes in *T. thermophila* cell viability, indicating low acute lethality of PSNPs toward this organism [32]. The average fluorescence intensity of ≥500 cells was then measured using flow cytometry to study the bioaccumulation kinetics of PSNPs in *T. thermophila*. As expected from the biokinetic modeling (with an r^2^ value > 0.9), the accumulation of PSNPs in the cells increased linearly with exposure time (Figure 2b) [33]. However, *T. thermophila* at different growth stages showed significant differences in the accumulation amount and rate of NPs. The accumulation of PSNPs in cells increased linearly with exposure time (r^2^ > 0.9 for all phases), supporting the use of a pseudo-first-order kinetic model to compare relative uptake rates between growth phases (Section 2.3). The highest rate of PSNP uptake occurred during the lag phase (cell density 1–6 × 10^4^ cells/mL), with 4 × 10^4^ cells/mL reaching 14.7 signal/cell/h, which is about 1.2–5.8 times the rate observed during the exponential phase (1–5 × 10^5^ cells/mL) and 7.7 times the rate observed during the stationary phase (>5 × 10^5^ cells/mL) (Figure 2c). In addition, the adsorption of PSNPs by *T. thermophila* cells varies at different growth stages. According to Figure 2b, when the cell density of *T. thermophila* was 4 × 10^4^, 4 × 10^5^, and 1 × 10^6^ cells/mL, the adsorption of PSNPs was 9.1, 12.7, and 11.5 signal/cell, respectively. The adsorption of PSNPs by *T. thermophila* at different growth stages was normalized to unit surface area (with cell dimensions approximated to a spherical model) to calculate the adsorption capacity per unit area. The results indicate that the normalized adsorption levels across all three growth phases were approximately 0.008 signal/cell/μm^2^, suggesting that the observed differences in total adsorption are primarily attributable to variations in cell size. Figure 2d–f provide direct visual evidence of the bioaccumulation and adsorption of PSNPs in *T. thermophila* at different growth phases. In lag-phase cells, multiple sites of colocalization between PSNPs and membrane structures were observed (Figure 2d), indicating substantial internalization of PSNPs via phagocytosis, which aligns with the accumulation kinetics observed experimentally. Additionally, isolated PSNP signals (green) were detected on the cell surface or within the cytoplasm across all three growth phases, suggesting that, in addition to oral apparatus-mediated phagocytosis, PSNPs may also enter *T. thermophila* cells through membrane transporter-mediated pathways (Figure 2e,f). While previous studies on other nanoparticles (e.g., Fe_2_O_3_ and TiO_2_ nanoparticles) have confirmed that *T. thermophila* can accumulate nanoparticles via both oral apparatus and protein-mediated endocytosis, those investigations primarily attributed the primary endocytic pathway to the type of nanoparticle [34,35]. However, they largely overlooked the intrinsic physiological and biochemical variations in the organism itself. Our results demonstrate that the endocytic rate and expression of phagocytosis-related proteins may vary significantly across different growth stages of *T. thermophila*. Meanwhile, proteomic and transcriptional profiling across distinct growth phases of *T. thermophila* confirms that the selection of three representative cell densities (4 × 10^4^, 4 × 10^5^, and 1 × 10^6^ cells/mL) effectively minimized inter-phase overlap, thereby ensuring that the observed uptake differences reflect genuine physiological state-dependent trends.

The accumulation rate and adsorption differences in PSNPs during the growth stages of *T. thermophila* may be attributed to several mechanisms. On one hand, the sensitivity to the toxicity of PSNPs might vary across different growth stages of *T. thermophila*. For example, Heng et al. demonstrated that zinc oxide nanoparticles exhibit lower cytotoxicity to human cells at high cell densities, leading to higher cell survival rates, while they are more toxic at lower densities. Mechanistically, changes in cell morphology indicate that high-density cells show greater tolerance due to reduced metabolism, decreased nanoparticle sedimentation, and increased cell-to-cell contact [36]. On the other hand, these differences could be related to the metabolic and proliferative states at different cell densities. For example, previous studies have shown that the expression of the *T. thermophila* membrane transport protein SOR and the gene GRL, which encodes the mucocyst cargo protein, is related to the growth cycle of *T. thermophila*. The mucocysts rapidly expel their contents through a squeezing mechanism that involves fusion with the membrane, playing a role in regulating exocytosis [37]. In summary, it is necessary to further clarify the physiological and biochemical characteristics of *T. thermophila* at different growth stages, as well as its toxicity sensitivity during PSNP exposure.

### 3.3. Antioxidant Capacity of T. thermophila at Different Growth Stages

**Figure 3:**
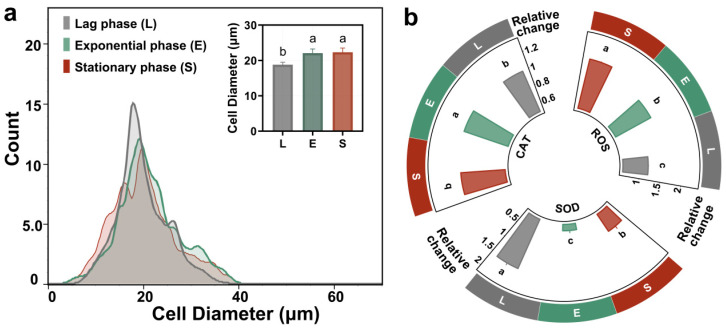
Physiological and biochemical indicators of *T. thermophila* cells at different growth stages. The cell size distribution of *T. thermophila* during the lag, exponential, and stationary phases, different lowercase letters (a, b, c) above the error bars indicate significant differences among groups (*p* < 0.05) (**a**). The relative concentration changes in intracellular ROS, SOD and CAT of the *T. thermophila* after 1 h exposure to 10 mg/L of PSNPs (**b**).

Figure 3a presents a correlated analysis of morphological and physiological changes in bacterial cells across the distinct growth phases of lag, exponential, and stationary. The histogram in Figure 3a reveals that the cell diameter distribution shifts from a monomodal peak during the lag phase to a broader, more complex distribution in the subsequent phases, with the inset bar chart quantitatively confirming that the average cell diameter is significantly larger during the exponential and stationary phases compared to the lag phase, as indicated by the statistical groupings (a, b). The surface-area-to-volume ratio (SA:V), a key determinant of a cell’s efficiency in nutrient exchange, was calculated by modeling cells at different growth stages as spheres. The lag-phase cells exhibited the highest SA:V ratio (0.319 μm^−1^), significantly greater than that of the exponential (0.271 μm^−1^) and stationary-phase cells (0.269 μm^−1^). This inverse relationship between cell size and SA:V ratio indicates that smaller cells are equipped with a greater membrane area relative to their volume, thereby enhancing their capacity for material exchange [38]. Consistent with the findings reported by Wang et al., although larger cells exhibit higher total accumulation, smaller cells demonstrate a greater accumulation rate [39]. According to their study, the Young’s modulus (elastic modulus) of cells increases significantly with larger cell size, indicating greater membrane stiffness and reduced deformability, which consequently leads to lower endocytic efficiency. In the present study, PSNP uptake in *T. thermophila* was higher during the exponential and stationary phases than during the lag phase only within the initial 0.5 h (Figure 3a). This might be attributed to a less pronounced difference in cell size between these growth phases compared to the 20–80 μm range reported by Wang et al. In addition, the potential toxic effects of PSNPs should be considered, as *T. thermophila* at different growth stages may exhibit differential susceptibility to these nanoparticles.

Multiple studies have confirmed that a key toxicological feature of smaller PSNPs was the induction of cellular oxidative stress [40]. Upon internalization, PSNPs disrupt the mitochondrial electron transport chain (ETC); impairment of the ETC leads to excessive generation of ROS, primarily superoxide anions, which overwhelms the cellular antioxidant capacity and results in ROS accumulation [41]. According to Figure 3b, after 1 h of PSNP exposure, the intracellular ROS levels in exponential and stationary phase *T. thermophila* cells were 1.5-fold and 1.8-fold higher than their respective blank controls, while the lag phase cells showed only a 1.2-fold increase. This indicates severe oxidative stress in exponential and stationary phase cells. It is noteworthy that although exposure to PSNPs induced short-term ROS accumulation in exponential and stationary phase Tetrahymena, the oxidative stress may be reversible over extended periods, as no significant apoptosis was observed after 24 h. This short-term measurement focuses on the immediate stress response during active PSNP uptake, which is most relevant to bioaccumulation mechanisms. Concurrently, lag phase cells upregulated SOD expression, whereas SOD levels in exponential and stationary phase cells were only 0.28-fold and 0.66-fold of their respective controls. As the primary enzyme in the antioxidant defense system, SOD converts highly reactive superoxide anions (^·^O_2_^−^) into less reactive hydrogen peroxide (H_2_O_2_). The decrease in SOD in exponential and stationary phase cells suggests a collapse of their primary antioxidant defense. In contrast, CAT, a subsequent enzyme in the antioxidant pathway, showed minimal changes compared to controls across all growth phases. CAT is responsible for decomposing H_2_O_2_ into harmless water and oxygen [42]. The stable CAT levels in exponential and stationary phase cells may be attributed to excessive consumption of SOD, leading to O_2_^−^ accumulation rather than H_2_O_2_ production. Meanwhile, the inherently lower ROS levels in lag phase cells may not necessitate altered CAT synthesis. Consistent with our findings, multiple studies have reported that nanoparticles exhibit greater toxicity toward exponentially growing cells than stationary phase cells. This phenomenon may stem from the increased contact of cells with nanoparticles, as stationary phase cells produce secretions that make it easier for nanoparticles to aggregate and settle, thereby reducing effective exposure [43,44]. However, our study did not observe significant PSNP aggregation under the experimental conditions investigated. This discrepancy may be attributed to the fact that while the cells for this study were collected based on growth stages defined by different cell densities, they were standardized to an equal density at the onset of the uptake experiment. Our experimental observation is supported by the findings of Zhang et al., who reported that the growth stage at which *T. thermophila* cells were harvested (e.g., early, mid, or late exponential phase, or stationary phase) did not significantly affect the total amount of organic matter they secreted in Dryl’s medium [17]. Therefore, the different oxidative stress responses of cells in different growth cycles may be derived from their inheritance of physiological and ecological changes in the cycle.

### 3.4. Proteomic and Transcriptomic Analysis of T. thermophila at Different Growth Stages

**Figure 4:**
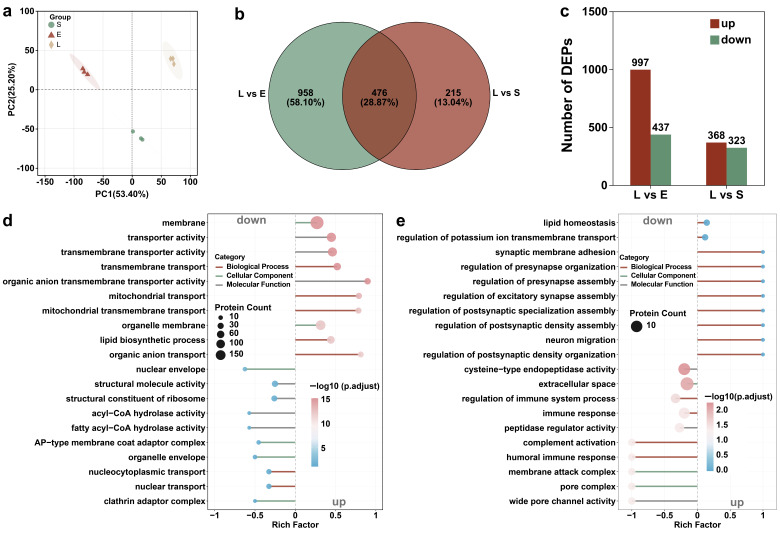
Proteomicanalysis of *T. thermophila* at different growth stages (L, E, and S respectively represent the lag phase, the exponential phase, and the stationary phase.). PCA score plots of protein expression profiles in *T. thermophila* across growth phases (**a**). Venn diagram of the differential proteins in *T. thermophila* cells at different growth stages (**b**). The number of differentially expressed proteins in lag-phase *T. thermophila* compared to those in the exponential and stationary phases (**c**). The top 10 significantly enriched differentially expressed proteins’ GO pathways (including 10 upregulated and 10 downregulated proteins): (**d**) Lag phase vs. Exponential phase, (**e**) Lag phase vs. Stationary phase (*p*-value < 0.05). The y-axis represents the GO pathways. The enrichment factor indicates the ratio of differentially expressed proteins in a given pathway to the total number of proteins annotated in that pathway. The size of the bubbles in the figure represents the abundance of proteins enriched in each respective pathway. (Protein FDR ≤ 0.01, Peptide FDR ≤ 0.01, Peptide Confidence ≥ 99%, XIC width ≤ 75 ppm).

Proteomic analysis of *T. thermophila* across different growth stages revealed that the differentially expressed proteins were predominantly annotated to GO terms such as cellular process, cellular anatomical structure, and catalytic activity (under molecular function) (Figure S2 and Figure 3). Principal component analysis revealed clear separation of protein expression patterns among the lag, exponential, and stationary phases, with PC1 and PC2 explaining 53.40% and 25.20% of the total variance, respectively (Figure 4a). The findings demonstrate that the growth phase serves as a major determinant of differential protein expression. The study by Miao et al. is consistent with our findings, demonstrating significant differences in upstream gene regulation across different growth stages of *T. thermophila* [45]. During the growth phase, 91 genes were specifically expressed and 155 genes were strongly upregulated (expression levels > 250-fold above the corrected background). These genes are primarily involved in cell division, metabolism, and essential cellular functions. In the starvation phase, 90 genes were specifically expressed and 616 genes were upregulated. Starvation induces cells to enter a conjugation-ready state, involving nutrient stress response and developmental transition [45]. As the upstream regulator of protein expression, genes indicate that stage-specific physiological processes are supported by distinct protein functions, such as the endocytosis of PSNPs.

Figure 4b presents a Venn diagram illustrating the distribution of differentially expressed proteins (DEPs) among the growth phase comparisons. The analysis identified 476 DEPs common to both the lag phase versus exponential phase and lag phase versus stationary phase comparisons. Furthermore, 958 and 215 DEPs were uniquely associated with the lag phase vs. exponential phase and lag phase vs. stationary phase comparisons, respectively. Comparative analysis showed 997 DEPs when comparing the lag phase with the exponential phase, and 368 DEPs when comparing the lag with the stationary phase (Figure 4c). This result indicates that lag-phase cells exhibit a more pronounced difference in protein expression when compared to exponential-phase cells, but show greater similarity to stationary-phase cells (Figure S4). This similarity in physiological state may be attributed to the fact that cells in the lag phase, having previously been in a nutrient-deprived environment, resemble those in the stationary phase. According to Mou et al., the increase in pCO_2_ reduced the ratios of particulate organic carbon (POC) to chlorophyll a (Chl a) and particulate organic nitrogen (PON) to Chl a in *Synechococcus* sp. PCC 7002 during the lag and stationary phases, while increasing these ratios during the exponential phase. These findings imply that the nutritional behaviors in the lag and stationary phases are more similar to each other [46].

The significant upregulation of pathways related to membrane transporter activity and organic anion transmembrane transporter activity suggests that lag-phase cells are actively modulating nutrient uptake and ion exchange to adapt to their early growth environment (Figure 4d). The enrichment of mitochondrial transport functions further indicates a heightened demand for metabolic energy and mitochondrial biogenesis, likely to support the upcoming exponential growth phase by boosting ATP production and optimizing oxidative phosphorylation efficiency. This active nutrient uptake behavior is an inducing factor for the significantly increased PSNP bioaccumulation rate in the lag phase of *T. thermophila* compared to other growth stages. Additionally, according to the research of Wei et al., proteins at the membrane interface (such as CDH1) directly regulate endocytic efficiency by affecting the kinetics of particle binding and vesicle formation [47]. Figure 4d demonstrates that, compared to the exponential period, a large number of upregulated differentially expressed proteins in lag phase *T. thermophila* are involved in the “membrane” pathway of the GO analysis. This includes membrane proteins, organelle membrane proteins, and proteins synthesized for the cytoplasmic membrane. Therefore, the upregulation of pathway proteins related to the membrane and transmembrane transporter activity is the direct factor responsible for the highest PSNP bioaccumulation rate in lag phase *T. thermophila* [48]. This aligns with the metabolic transition from growth-driven biosynthesis in exponential phase to a more quiescent, resource-conserving state in lag phase. The downregulated protein expression associated with structural molecular activity and ribosomal functions indicates that exponential-phase cells are primed for biomass accumulation and division, rather than the substantial uptake of nutrients, including PSNPs (Figure 4d). Compared to the contrast between the lag and exponential phases, the lag phase exhibited enhanced stress adaptation, signal transduction, and environmental sensing when compared to the stationary phase (Figure 4e). Conversely, the downregulation of peptidase regulator activity, complement activation, and membrane attack complex formation suggests suppressed protein degradation and immune effector mechanisms in the lag phase. This indicates a resource allocation favoring stress adaptation. In contrast, the stationary phase showed insufficient stress adaptation, which aligns with our previous observation of weak antioxidant capacity in *T. thermophila* during this phase (Figure 3b).

These findings demonstrate a clear stage-specific proteome reprogramming in *T. thermophila* that correlates with the bioaccumulation of PSNPs. The lag phase was marked by a pronounced emphasis on transport and metabolic preparedness, enabling the rapid internalization of PSNPs. Exponential phase cells prioritized biomass accumulation and division, whereas the stationary phase was characterized by enhanced expression of proteins involved in signaling and environmental perception, indicating a greater emphasis on survival and maintenance.

### 3.5. Identification of Key Functional Proteins

**Figure 5:**
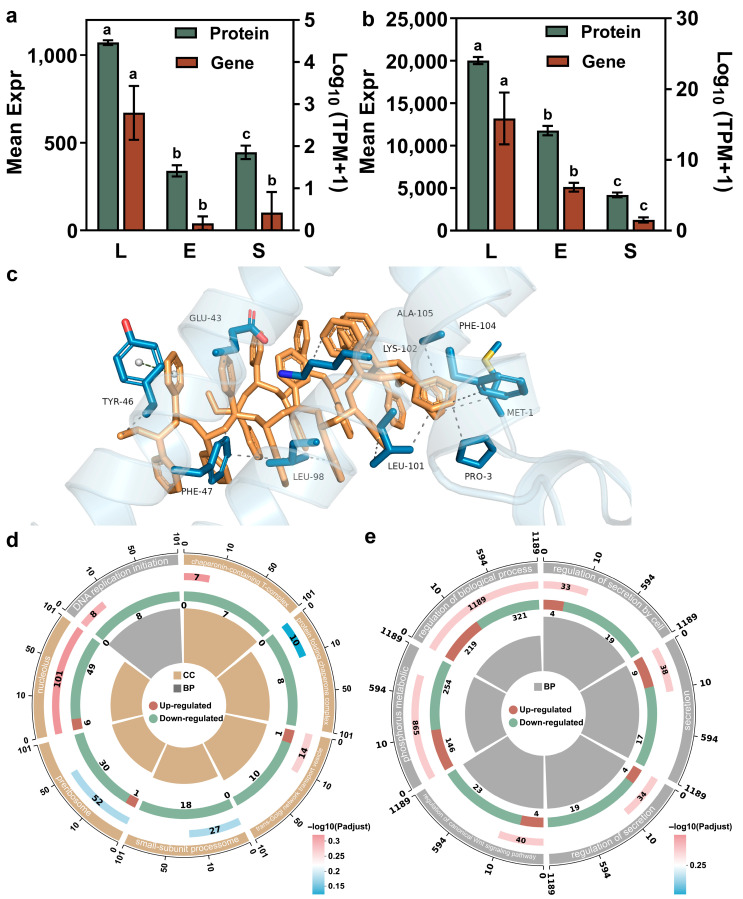
Keyproteinsinvolved in PSNP internalization and the stress response in *T. thermophila*. Relative expression of transmembrane protein (accession: XP_001009830.2) across the three growth phases of *T. thermophila*, different lowercase letters (a, b, c) above the error bars indicate significant differences among groups (*p* < 0.05) (**a**). Relative expression of an FAD/FMN-binding family oxidoreductase (accession: XP_977198.1) across the three growth phases of *T. thermophila* (**b**). Molecular view of the interaction interface (**c**). Protein residues are shown in slate blue sticks; the R-group molecule is shown in yellow sticks. Hydrophobic interactions and π-π stacking are indicated by gray and seafoam green dashed lines, respectively. Downregulated pathways among the top 30 significantly enriched pathways in the comparison of gene expression differences between Lag phase vs. Exponential phase (**d**), Lag phase vs. Stationary phase (*p*-value < 0.05) (**e**). The enrichment circle diagram consists of four concentric rings, arranged from outer to inner: First Ring: Enriched categories, with the outer ring representing the scale for the number of genes. Different colors represent different categories. Second Ring: The number of genes in the background for each category, along with Q-values or *p*-values. The longer the bar, the more genes are present; the redder the color, the smaller the *p*-value, indicating greater enrichment significance. Third Ring: A bar graph representing the proportion of upregulated and downregulated genes, with the specific values displayed at the bottom. Fourth Ring: The Rich Factor values for each category (the number of differentially expressed genes in the category divided by the number of genes in the background).

Among the shared pool of upregulated differential proteins common to both the lag vs. exponential and lag vs. stationary comparisons, the carrier protein displayed the most statistically significant change in expression. According to Figure 5a, the carrier protein of *T. thermophila* has the highest expression level during the lag phase, while its expression levels significantly decrease during the exponential phase and stationary phase. This temporal expression pattern is highly consequential for explaining the previously observed peak in nanoparticle (e.g., PSNP) bioaccumulation during the lag phase. Carrier proteins are integral to active transport processes across cell membranes. The pronounced upregulation of this protein in the lag phase suggests a state of heightened nutrient import and metabolic preparation as the cells exit dormancy. This proactive physiological state likely facilitates the non-specific internalization of environmental particles, including nanoparticles, via enhanced endocytic activity or other carrier-mediated transport pathways. The molecular docking results support our hypothesis. Figure 5c and Table S3 illustrate the interactions between the protein and the molecule, which represents the PSNP surface using a 20-mer polystyrene oligomer. The data reveal 15 hydrophobic interactions and one π-π stacking interaction between the protein and the ligand. The presence of these multiple favorable interactions, as revealed by molecular docking, suggests that the binding process is spontaneous. Thus, the peak expression of this transporter protein provides a direct mechanistic link to the highest bioaccumulation rate observed in lag-phase cells, by initiating the internalization of PSNPs upon receiving particle signals.

In addition to proteins with uncharacterized functions, an FAD/FMN-binding family oxidoreductase was also significantly upregulated in lag phase *T. thermophila*. According to Figure 5b, the expression of FAD/FMN-binding family oxidoreductase is the highest in the lag phase, drops significantly in the exponential phase, and reaches the absolute lowest level in the stationary phase. This profile aligns precisely with the postulated antioxidative capacity of the cells throughout their growth cycle. Proteins in this family are pivotal components of cellular antioxidant defense systems, involved in redox homeostasis and the neutralization of ROS [49]. The maximal expression of this oxidoreductase in the lag phase indicates a robust preparatory antioxidant defense, potentially to manage ROS generated during the metabolic reactivation and rapid membrane remodeling characteristic of this stage. Conversely, the significantly lowest expression in the stationary phase reflects a diminished investment in antioxidative processes, consistent with the overall metabolic downregulation and reduced metabolic activity that defines the stationary phase. This correlation strongly suggests that the changing expression of this oxidoreductase is a key factor underlying the dynamic antioxidant capacity, which is strongest in the lag phase and weakest in the stationary phase. This was consistent with the phenomenon observed in Figure 3b, where the ROS accumulation was the highest in the stable phase of the cells.

Consistent transcriptomic and proteomic data further validated the gene regulation-based physiological changes in *T. thermophila* at different growth stages. Based on the GO enrichment analysis of downregulated pathways in the exponential and stationary phases compared to the lag phase (Figure 5d,e), the reduced accumulation rate of PSNPs during the exponential and stationary phases can be attributed to the significant downregulation of key biological processes related to cellular transport and energy metabolism. Specifically, the downregulation of transport-related pathways suggests diminished activity in vesicle trafficking, secretory pathways, and ribosomal biogenesis. This likely impaired the efficiency of endocytosis and intracellular transport mechanisms responsible for PSNP uptake and localization. Concurrently, the suppression of energy-metabolic processes indicates reduced ATP production and metabolic activity [50]. Since PSNP internalization is energy-dependent, limited energy availability further contributed to slower accumulation during the exponential and stationary phases. In contrast, the robust expression of these pathways during the lag phase supported higher endocytic activity and energy supply, facilitating more rapid PSNP accumulation. Thus, the phase-dependent decline in transport and metabolic capacity aligns with the observed reduction in PSNP uptake rates.

In summary, the proteomic data provide compelling evidence at the molecular level: the differential expression of specific functional proteins, such as the carrier protein and the FAD/FMN-binding oxidoreductase, directly underpins the distinct physiological states of *T. thermophila* at each growth phase, thereby explaining the observed phenomena of nanoparticle bioaccumulation and antioxidant strength.

## 4. Conclusions

**Figure 6:**
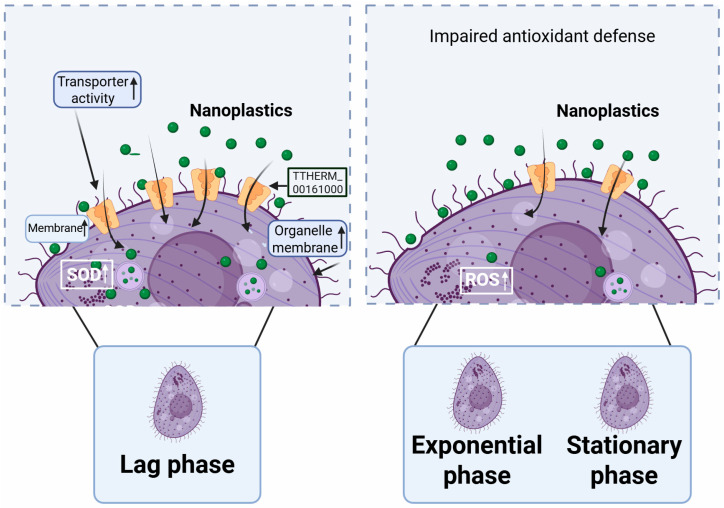
Mechanisms underlying the differential accumulation rates of PSNPs in *T. thermophila* across distinct growth phases.

This study reveals a pronounced growth-phase-dependent bioaccumulation of PSNPs in *T. thermophila*, with the lag phase exhibiting the highest uptake rate—1.2–5.8 times and 7.7 times greater than that in the exponential and stationary phases, respectively. The underlying mechanism for this phenomenon is attributed to a unique “metabolically primed” state of lag phase cells, characterized by a larger surface area-to-volume ratio and critical proteomic reprogramming. The significant upregulation of specific proteins, notably a carrier protein and an FAD/FMN-binding oxidoreductase, collectively enhances transmembrane transport capacity and antioxidant defense (Figure 6). This physiological state inadvertently facilitates the efficient internalization of PSNPs during active nutrient foraging. These findings carry significant environmental implications, suggesting that traditional risk assessments based on exponential phase cells may systematically underestimate the actual bioaccumulation risk of NPs in natural water bodies, where microorganisms often exist under nutrient-limited conditions analogous to the lag or stationary phases. It is, therefore, imperative to incorporate such physiological dynamics into risk assessment frameworks to accurately predict the environmental behavior and ecological impacts of PSNPs. Furthermore, to precisely control experimental conditions and elucidate underlying mechanisms, this study utilized well-dispersed PSNPs in a defined Dryl’s medium. However, in natural aquatic environments, NP surfaces can rapidly adsorb environmental natural organic matter (NOM, e.g., humic acids, fulvic acids), proteins, and other biomolecules, forming an “eco-corona”. Given this limitation, a highly valuable future research direction involves investigating how the eco-corona modulates the accumulation kinetics and molecular mechanisms of NPs in microorganisms. Such research would not only enhance the environmental relevance of the findings but also deepen our understanding of the interactions at the NP–bio interface.

## Data Availability

The original contributions presented in this study are included in the article/Supplementary Material. Further inquiries can be directed to the corresponding author.

## Supplementary Materials

The following supporting information can be downloaded at: https://www.mdpi.com/article/10.3390/antiox14121456/s1, Figure S1: Viability of *T. thermophila* across growth phases after 24 h exposure to PSNPs. Cell counts of (a) lag, (b) exponential, and (c) stationary phase cells exposed to a concentration gradient of PSNPs (0, 0.1, 1, 10, and 100 mg/L) for 24 h; Figure S2: Functional enrichment of DEPs (lag vs. exponential phase) in *T. thermophila*. Significantly enriched GO pathways among (a) up-regulated and (b) down-regulated protein sets are shown; Figure S3: Functional enrichment of DEPs (lag vs. stationary phase) in *T. thermophila*. Significantly enriched GO pathways among (a) up-regulated and (b) down-regulated protein sets are shown; Figure S4: Volcano plot analysis of DEPs. (a) Lag vs. exponential phase. (b) Lag vs. stationary phase. Protein expression changes (log2 fold change, x-axis) are plotted against their statistical significance (-log10 FDR, y-axis). Each point denotes a single protein; Figure S5: (a) Up- and down-regulated differentially expressed genes (DEGs) in *T. thermophila* during exponential and stationary phases compared to the lag phase. (b) PCA score plots of DEGs in *T. thermophila* across growth phases Table S1: Composition of SPP (Super Proteose Peptone) medium; Table S2. Composition of Dryl’s medium; Table S3. The interaction situation between proteins and molecules. Text S1. Cytotoxicity assays. Text S2. Proteomics analysis. Text S3. Transcriptomics analysis.
