# Supplementary material for: Mechanism of Growth Phase-Dependent Nanoplastic Bioaccumulation in Tetrahymena thermophila
Source: Antioxidants (Basel). 2025 Dec 4;14(12):1456. doi: 10.3390/antiox14121456 (PMC12729904; doi:10.3390/antiox14121456)
Supplement: Supplementary file 1 [file antioxidants-14-01456-s001.zip › antioxidants-4012810-supplementary.pdf]

## Supporting Information

# Mechanism of growth phase-dependent nanoplastic bioaccumulation in *Tetrahymena thermophila*

Zhongquan Jiang [1,2], Tianyi Wei [2], Haipeng Tong [2], Ruikai Xing [2], Di Peng [1], Tao Yuan [2], Ling Zhao [2], Minghua Min[1,3,*] and Wenbo Guo [2,4*]

[1] East China Sea Fisheries Research Institute, Chinese Academy of Fishery Sciences, Shanghai 200090, China; zhongquanj@sjtu.edu.cn (Z.J.); pengdi@ecsf.ac.cn (P.D.); minmh@ecsf.ac.cn (M.M.)

[2] Key Laboratory of Environmental Health Impact Assessment of Emerging Contaminants, Ministry of Ecology and Environment, School of Environmental Science and Engineering, Shanghai Jiao Tong University, Shanghai 200240, China; sardine0722@sjtu.edu.cn (T.W.); tong-arabidopsis@sjtu.edu.cn (H.T.); xrk382937427@sjtu.edu.cn (R.X.); taoyuan@sjtu.edu.cn (T.Y.); wszhaoling@sjtu.edu.cn (L.Z.); dg1825010@smail.nju.edu.cn (W.G.)

[3] Zhoushan Putuo Deep Blue Fishery Technology Research and Development Center, Zhoushan 316100, China; minmh@ecsf.ac.cn (M.M.)

[4] International Research Center for Marine Biosciences, Shanghai Ocean University, Ministry of Science and Technology, College of Fisheries and Life Science, Shanghai 201306, China

[*]Correspondence: minmh@ecsf.ac.cn (M.M.); dg1825010@smail.nju.edu.cn (W.G.)

This supplementary material includes 16 pages, 3 Texts, 3 Tables, and 5 Figures.

## Contents

**Text S1. Cytotoxicity assays.**

Briefly, *T. thermophila* cells from three distinct growth phases (lag, exponential, and stationary) were exposed to 10 mg/L polystyrene nanoparticles (PSNPs) for 1 h, with a control group without PSNPs included for each phase. At the end of exposure, cells from each group were collected by centrifugation at 3000 rpm for 5 min, washed three times with fresh Dryl's medium, and resuspended in 100 μL of DCFH-DA at a working concentration of 10 μmol/L. After incubation in the dark at 37 °C for 20 min, the cells were washed again with Dryl's medium to remove excess probe. Fluorescence intensity was measured using a microplate reader (SpectraMax iD3, Molecular Devices) with excitation and emission wavelengths set at 488 nm and 525 nm, respectively. The relative ROS level for each growth phase was expressed as the ratio of the fluorescence intensity of the PSNP-exposed group to that of the corresponding control. All measurements included blanks (no sample) and controls (no PSNPs). The ROS levels were calculated from data corrected by subtracting the blank values.

The activities of superoxide dismutase (SOD) and catalase (CAT) in *T. thermophila* cells at three growth phases after exposure to 10 mg/L PSNPs were determined using commercial assay kits (Beyotime Biotechnology Inc., Shanghai, China), following the manufacturer's instructions.

For SOD activity measurement, the cell supernatant was incubated with the detection reagent at 37 °C for 30 min, similar to the ROS assay procedure. Absorbance

was measured at 560 nm using a microplate reader. SOD activity for each treatment group was calculated as follows:

Inhibition percentage = $(Ab_1 - A_s) / (Ab_1 - Ab_2) \times 100\%$

SOD enzyme activity (units) = Inhibition percentage / (1 − Inhibition percentage)

where $Ab_1$, $A_s$, and $Ab_2$ represent blank control 1, sample absorbance, and blank control 2, respectively.

Catalase (CAT) activity in *T. thermophila* cells at lag, exponential, and stationary growth phases following exposure to 10 mg/L PSNPs was determined by incubating the supernatant with detection reagent at 25 °C for 15 min and measuring the absorbance at 520 nm. Total protein content in the solution was determined using the Coomassie Brilliant Blue method, and CAT activity was calculated using formula S1.

$$\text{CAT enzyme activity (units/mg)} = \frac{M_b - M_s}{T \times V \times C} \qquad \text{(S1)}$$

Here, $M_b$ and $M_s$ represent the residual hydrogen peroxide in micromoles for the blank control and the sample, respectively, which were calculated using the standard curve. T, V, and C denote the reaction time, sample volume, and total protein concentration in the sample, respectively.

**Text S2. Proteomics analysis.**

1. Total protein extraction

Take out the samples in the frozen state and put them on ice. The samples were suspended in protein lysis buffer (8M urea, 1% SDS) which included appropriate protease inhibitor to inhibit protease activity and the mixture were treated by a high-flux tissue grinding machine for 3 times, 180s each. Then, the non-contact cryogenic sonication was performed for 30 min. After centrifugation at 16000 g at 8 °C for 30 min, the concentration of protein from the supernatant collected was determined by Bicinchoninic acid (BCA) method by BCA Protein Assay Kit (Thermo Scientific). Protein quantification was performed according to the kit protocol. The precipitate was dissolved in a protein lysate solution containing 8M urea, 1% SDS, and a protease inhibitor cocktail. After sonication on ice for 2 minutes and centrifugation at 12,000 g for 20 minutes at 4 °C, the protein content of 1 μL of the supernatant was determined using the ProteoAnalyzer (M5350AA).

2. Protein digestion

100 μg protein re-suspended with Triethylammonium bicarbonate buffer (TEAB) which with the final concentration of 100mM. The mixture was reduced with Tris (2-carboxyethyl) phosphine (TCEP) which with the final concentration of 10 mM at 37 °C for 60 min and alkylated with iodoacetamide (IAM) which with the final concentration of 40 mM at room temperature for 40 min in darkness. After centrifugation at 10000 g at 4 °C for 20 min, the pellet was collected, which re-suspended with 100 μL Triethylammonium bicarbonate buffer (TEAB) which with the final concentration of 100 mM. Trypsin was added at 1:50 trypsin-to-protein mass ratio and incubated at 37 °C overnight.

After trypsin digestion, the peptides were dried by vacuum pump. Then, the

enzymatically drained peptides were re-solubilized with 0.1% trifluoroacetic acid (TFA), and the peptides were desalted with HLB and dried by vacuum concentrator. Finally, the peptides were quantified using the NANO DROP ONE (Thermo Scientific) by UV absorption value.

Based on peptide quantification results, the peptides were analyzed by a VanquishNeo coupled with a timsTOF Ultra2 mass spectrometer (Bruker, Germany) at Majorbio Bio-Pharm Technology Co. Ltd. (Shanghai, China). Briefly, the uPAC High Throughput column (75 µm × 5.5 cm, Thermo, USA) was used with solvent A (water with 2% ACN and 0.1% formic acid) and solvent B (water with 80% ACN and 0.1% formic acid). The chromatography run time was set to 8 minutes. Data acquisition was managed by Compass HyStar software (Bruker, Germany). The timsTOF Ultra2 mass spectrometer was operated in DIA mode with a positive ion detection mode, and the ion source voltage was set to 4.5 kV. The mass spectrometry scanning range was 100-1700 m/z.

3. Protein identification

Spectronaut software (Version 19) was used to search the DIA raw data. The parameters are as follows: The peptide length range was set to 7-52; Enzyme cutting site was trypsin/P; The maximum missed cleavage site was 2; Carbamidomethylation of cysteines as fixed modification, and oxidation of methionines and protein N-terminal acetylation as variable modifications; Protein FDR ≤ 0.01, Peptide FDR ≤ 0.01, Peptide Confidence ≥ 99%, XIC width ≤ 75 ppm. The protein quantification method was MaxLFQ.

4. Statistical analyses

Bioinformatic analysis of proteomic data was performed with the Majorbio Cloud platform. P-values and Fold change (FC) for the proteins between the two groups were

calculated using R package “t-test”. The thresholds of fold change (>1.2 or <0.83) and P-value <0.05 were used to identify differentially expressed proteins (DEPs). Functional annotation of all identified proteins was performed using GO pathway. DEPs were further used for GO enrichment analysis. Protein-protein interaction analysis was performed using the String v11.5.

**Text S3. Transcriptomics analysis.**

1. RNA Extraction

Total RNA was extracted from the tissue using TRIzol® Reagent according to the manufacturer's instructions. RNA quality was assessed with the 5300 Bioanalyzer (Agilent) and quantified using the ND-2000 spectrophotometer (NanoDrop Technologies). Only high-quality RNA samples (OD260/280 = 1.8–2.2, OD260/230 ≥ 2.0, RIN ≥ 6.5, 28S:18S ratio ≥ 1.0, and quantity >1 μg) were used for sequencing library construction.

2. Library Preparation and Sequencing

RNA purification, reverse transcription, library construction, and sequencing were performed at Shanghai Majorbio Bio-pharm Biotechnology Co., Ltd. (Shanghai, China) in accordance with the manufacturer's protocols. The RNA-seq transcriptome library was prepared following the Illumina® Stranded mRNA Prep, Ligation kit (San Diego, CA) with 1 μg of total RNA. Briefly, mRNA was isolated via polyA selection using oligo(dT) beads and fragmented. First-strand cDNA was synthesized with random hexamer primers, followed by second-strand synthesis. The cDNA then underwent end repair, phosphorylation, and adapter ligation. Libraries were size-selected for cDNA fragments of 300–400 bp using magnetic beads and amplified through 10–15 PCR cycles. After quantification with Qubit 4.0, sequencing was conducted on either the NovaSeq X Plus platform (PE150) with the NovaSeq Reagent Kit or the DNBSEQ-T7 platform (PE150) with the DNBSEQ-T7RS Reagent Kit (v3.0).

3. Quality Control and Read Mapping

Raw paired-end reads were trimmed and quality-controlled using fastp with default parameters. Clean reads were aligned to the reference genome in orientation-aware mode with HISAT. The mapped reads for each sample were assembled using StringTie in a reference-based approach.

4. Differential Expression Analysis and Functional Enrichment

To identify differentially expressed genes (DEGs) between two sample groups, transcript expression levels were calculated as transcripts per million (TPM). Gene abundance quantification was performed with RSEM. Differential expression analysis was carried out using DESeq2 or DEGseq. DEGs were defined as those with $|\log_2 \text{fold change}| \geq 1$ and a false discovery rate (FDR) $< 0.05$ for DESeq2 or FDR $< 0.001$ for DEGseq. Functional enrichment analysis, including Gene Ontology (GO) and KEGG pathway analysis, was conducted to identify significantly enriched terms at a Bonferroni-corrected P-value $< 0.05$ relative to the whole transcriptome background. GO enrichment was performed with Goatools, and KEGG pathway analysis was implemented using Python scipy software.

5. Alternative Splicing Event Identification

All alternative splicing events were detected with the recently released program rMATS. Only isoforms that matched the reference sequence or contained novel splice junctions were considered. Splicing differences were categorized as exon inclusion, exclusion, alternative 5′ or 3′ splice sites, and intron retention events.

**Table S1.** Composition of SPP (Super Proteose Peptone) medium.

| Composition | Concentration |
|---|---|
| Proteose peptone | 2% (w/w) |
| Yeast extract | 0.1% (w/w) |
| Glucose | 0.2% (w/w) |
| Ferric citrate | 0.003% (w/w) |
| Penicillin G | 100 units/mL |
| Streptomycin sulfate | 100 μg/mL |
| Amphotericin B | 0.025 μg/mL |

**Table S2.** Composition of Dryl's medium.

| Composition | Concentration |
|---|---|
| $NaH_2PO_4 \cdot H_2O$ | 2 mM |
| $Na_2\ HPO_4$ | 1 mM |
| $CaCl_2$ | 1.5 mM |

**Table S3.** The interaction situation between proteins and molecules.

| **Index** | **Residue** | AA | Distance | Ligand Atom | Protein Atom |
|---|---|---|---|---|---|
| 1 | 1A | MET | 3.76 | 1307 | 5 |
| 2 | 3A | PRO | 3.32 | 1313 | 26 |
| 3 | 43A | GLU | 3.55 | 1360 | 441 |
| 4 | 46A | TYR | 3.71 | 1389 | 464 |
| 5 | 47A | PHE | 2.97 | 1364 | 484 |
| 6 | 47A | PHE | 3.67 | 1351 | 484 |
| 7 | 47A | PHE | 3.38 | 1387 | 482 |
| 8 | 98A | LEU | 3.31 | 1339 | 965 |
| 9 | 98A | LEU | 3.41 | 1351 | 965 |
| 10 | 101A | LEU | 3.66 | 1327 | 1006 |
| 11 | 101A | LEU | 3.38 | 1314 | 1007 |
| 12 | 102A | LYS | 3.83 | 1322 | 1015 |
| 13 | 102A | LYS | 3.57 | 1319 | 1014 |
| 14 | 104A | PHE | 3.96 | 1313 | 1037 |
| 15 | 105 A | ALA | 3.36 | 1311 | 1047 |

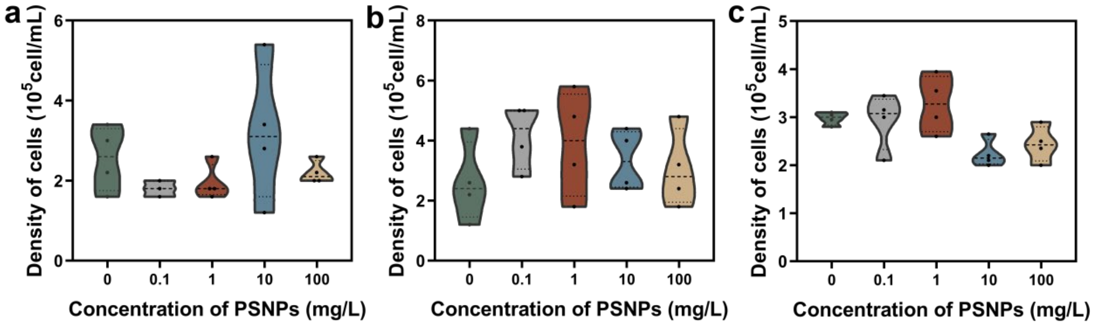


**Figure S1.** Viability of *T. thermophila* across growth phases after 24 hours exposure to PSNPs. Cell counts of (a) lag, (b) exponential, and (c) stationary phase cells exposed to a concentration gradient of PSNPs (0, 0.1, 1, 10, and 100 mg/L) for 24 hours.

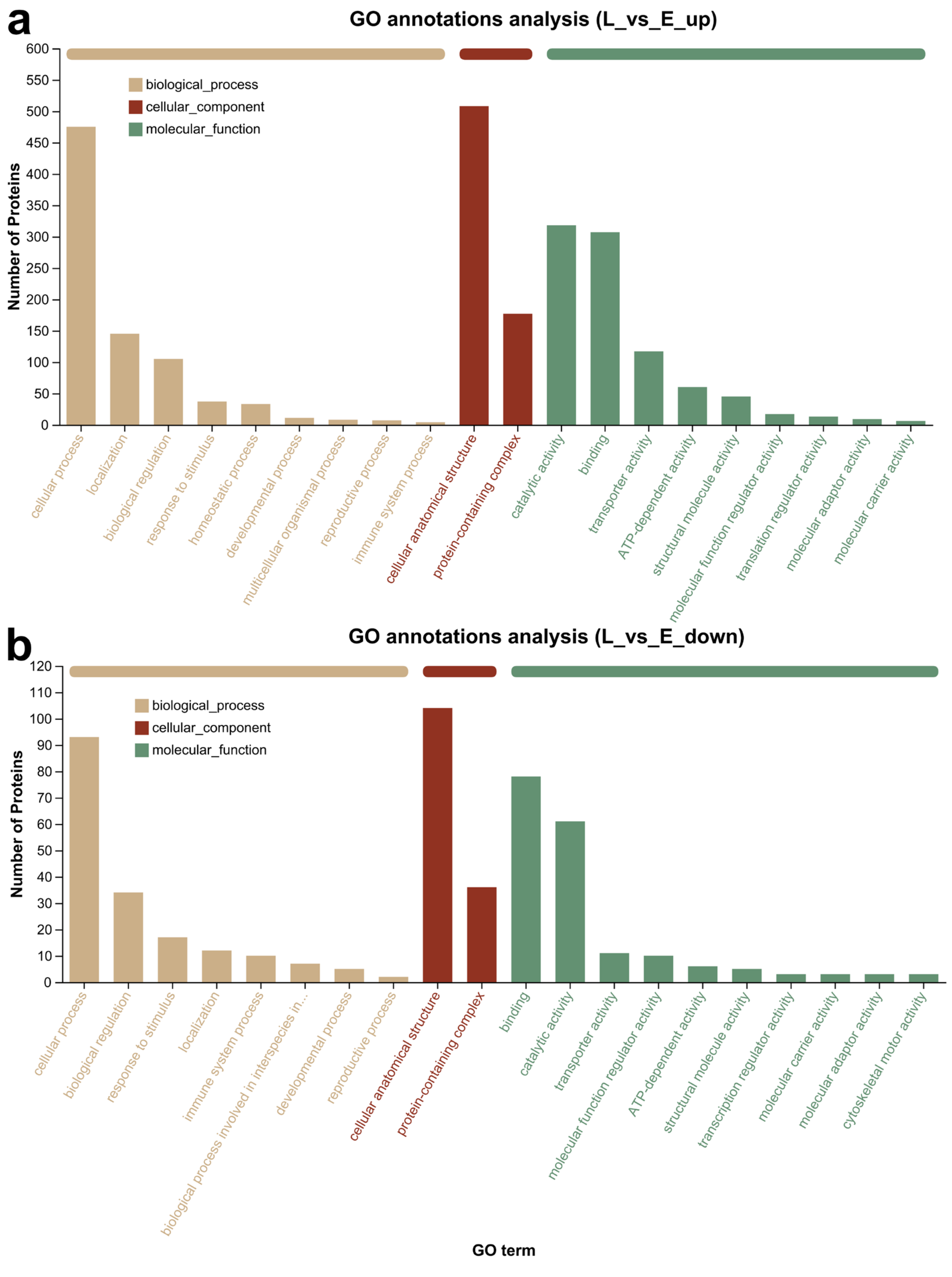


**Figure S2.** Functional enrichment of DEPs (lag vs. exponential phase) in *T. thermophila*. Significantly enriched GO pathways among (a) up-regulated and (b) down-regulated protein sets are shown.

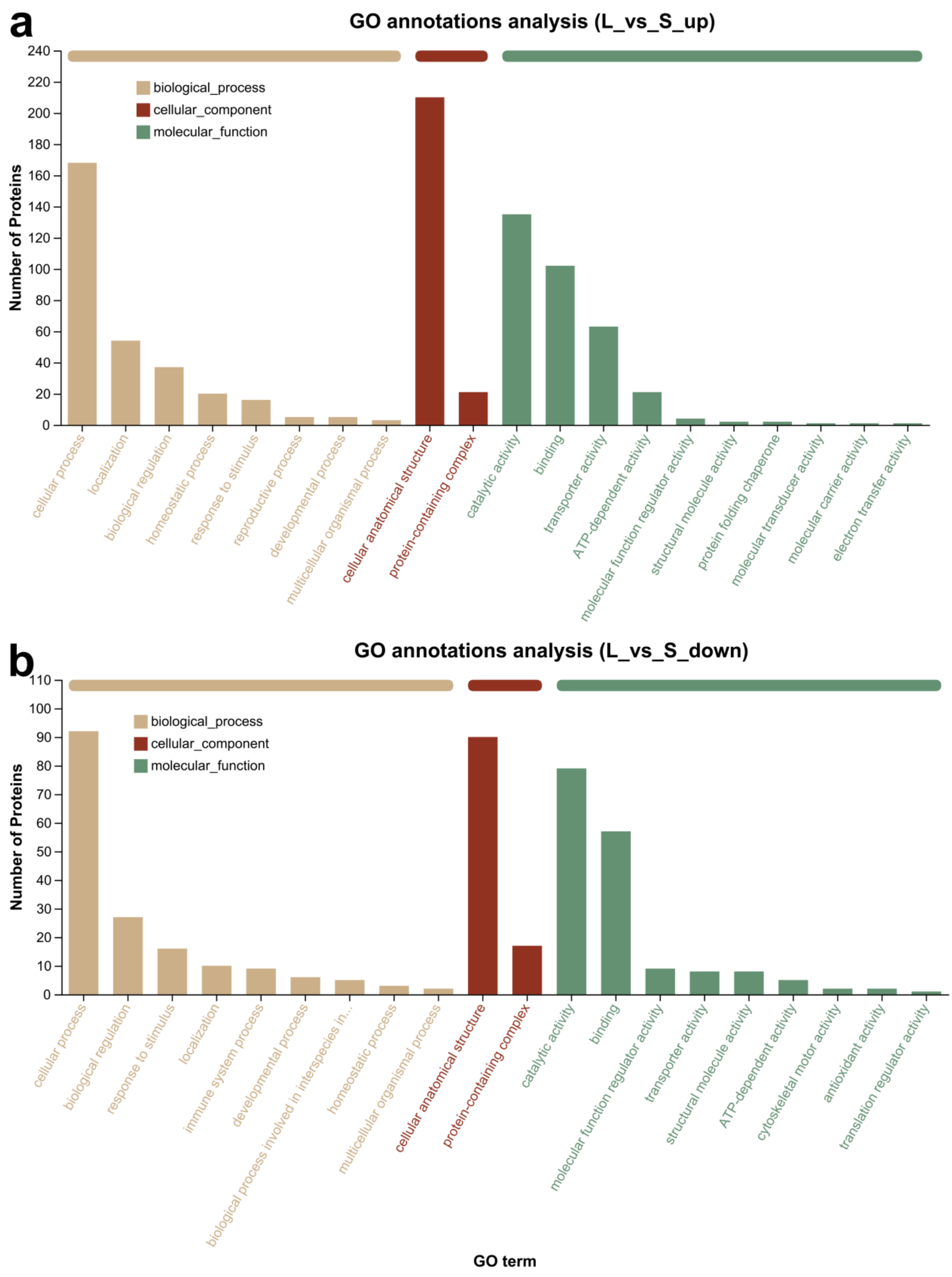


**Figure S3.** Functional enrichment of DEPs (lag vs. stationary phase) in *T. thermophila*. Significantly enriched GO pathways among (a) up-regulated and (b) down-regulated protein sets are shown.

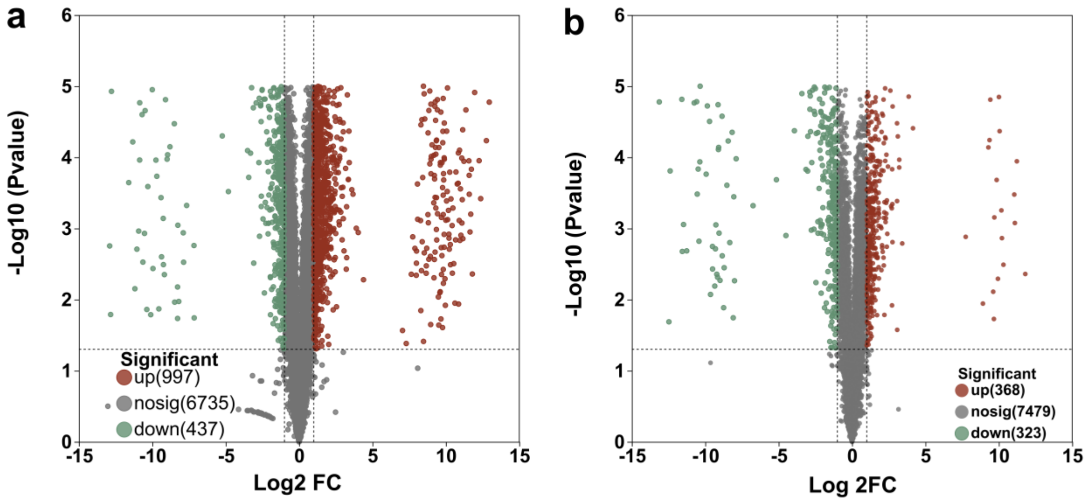


**Figure S4.** Volcano plot analysis of DEPs. (a) Lag vs. exponential phase. (b) Lag vs. stationary phase. Protein expression changes (log2 fold change, x-axis) are plotted against their statistical significance (-log10 FDR, y-axis). Each point denotes a single protein.

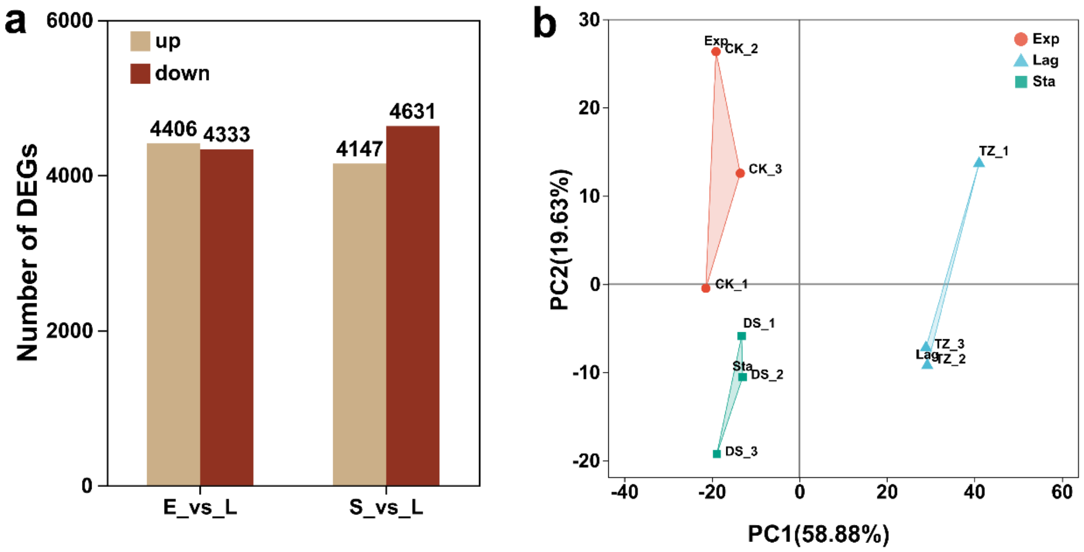


**Figure S5.** (a) Up- and down-regulated differentially expressed genes (DEGs) in *T. thermophila* during exponential and stationary phases compared to the lag phase. (b) PCA score plots of DEGs in *T. thermophila* across growth phases.
